# Anti-RGMa neutralizing antibody ameliorates vascular cognitive impairment in mice

**DOI:** 10.1016/j.neurot.2024.e00500

**Published:** 2024-11-29

**Authors:** Masaya Yamamoto, Takahide Itokazu, Hiroki Uno, Takakuni Maki, Nao Shibuya, Toshihide Yamashita

**Affiliations:** a Department of Molecular Neuroscience, Graduate School of Medicine, Osaka University, Suita, Japan; b Department of Neuro-Medical Science, Graduate School of Medicine, Osaka University, Suita, Japan; c Department of Neurology, Graduate School of Medicine, Kyoto University, Kyoto, Japan; d WPI-Immunology Frontier Research Center, Osaka University, Suita, Japan

**Keywords:** Chronic hypoperfusion, Neurogenesis, Cognitive impairment, Repulsive guidance molecule-a (RGMa)

## Abstract

Repulsive Guidance Molecule A (RGMa) is well-recognized for its role in axon guidance. Recent studies have unveiled its diverse functions under pathological conditions within the central nervous system, such as spinal cord injury, multiple sclerosis, and Parkinson's disease. In this study, we explored the involvement of RGMa and the therapeutic effects of an anti-RGMa neutralizing antibody in a mouse model of vascular dementia (VaD). The VaD mouse model was established using the bilateral common carotid artery stenosis (BCAS) method. Immunohistochemical analysis revealed that these mice exhibited increased RGMa expression in the hippocampus, which coincided with reduced neurogenesis and impaired cholinergic innervation. These alterations manifested as cognitive impairments in the BCAS mice. Significantly, treatment with anti-RGMa neutralizing antibody reversed these pathological changes and cognitive deficits. Our findings suggest that RGMa plays a pivotal role in VaD pathology within the hippocampus and propose the anti-RGMa antibody as a promising therapeutic avenue for treating VaD.

## Introduction

Chronic cerebral hypoperfusion (CCH), characterized by a moderate and persistent reduction in cerebral blood flow (CBF), is a major pathological factor in vascular dementia (VaD) [1,2], and has been identified as an independent risk factor for cognitive decline [3,4]. However, a considerable gap remains in our understanding of the VaD pathologies that are influenced by CCH, hampering the development of targeted therapies.

Notably, substantial evidence has indicated hippocampal atrophy in patients with VaD [5,6]. Due to its lower vascular density than the cortex [7], the hippocampus undergoes a significant reduction in blood flow in models of CCH [8], and this reduction in hippocampal blood flow has been implicated in hippocampal atrophy and cognitive decline [9,10]. However, the pathological characteristics of the hippocampus in VaD remain unclear.

Hippocampal neurogenesis involves the generation of new neurons from neural stem cells located in the subgranular zone (SGZ) of the dentate gyrus [11]. This process is fundamental to learning, memory, and emotional regulation, as highlighted by numerous studies [[12], [13], [14]]. Pathological conditions such as depression [15], Alzheimer's disease [16,17], and aging [18] are associated with a significant decline in this process, which leads, in part, to cognitive deficits. Given its importance, many studies have focused on modulating neurogenesis as a potential therapeutic strategy for neurodegenerative conditions [19]. However, our understanding of how CCH affects hippocampal neurogenesis and the underlying molecular mechanisms is limited.

Repulsive guidance molecule-a (RGMa), a glycosylphosphatidylinositol (GPI)-anchored protein, is implicated in many cellular processes. By interacting with its receptor, neogenin, RGMa inhibits neurite outgrowth and induces cell death, compromising the regenerative potential of the central nervous system (CNS) [[20], [21], [22], [23]]. The deleterious role of RGMa has also been reported in a range of CNS disorders, including multiple sclerosis [22,[24], [25], [26]], neuromyelitis optica spectrum diseases [27], cerebral infarction [[28], [29], [30]], spinal cord injury [[31], [32], [33]], and Parkinson's disease [34,35]. Considering its potential role in cognitive function, previous studies have demonstrated that neogenin is expressed in newborn neurons in the dentate gyrus of the adult hippocampus [36].

In the present study, we investigated whether RGMa serves as an inhibitory modulator of neurogenesis in VaD. We employed a bilateral common carotid artery stenosis (BCAS) mouse model and observed a pronounced increase in RGMa levels within the dentate gyrus. We observed disruptions in neurogenesis, deficits in cholinergic fibers, and a marked decline in cognitive function. Furthermore, we obtained evidence that RGMa is involved in hippocampal pathology, and the suppression of RGMa signaling effectively ameliorated BCAS-induced cognitive impairment.

## Materials and Methods

### Mice

All experimental procedures were approved by the Institutional Ethics Committee of Osaka University and conducted in strict compliance with the Osaka University Medical School Guidelines for the Care and Use of Laboratory Animals. Adult C57BL/6 ​J male mice, aged 8–10 weeks, were procured from CLEA Japan, Inc. The mice were housed under controlled conditions with a consistent 12-h dark/light cycle and provided ad libitum access to food and water.

### BCAS model establishment

We utilized the BCAS surgical procedure as outlined by Shibata et al. [37]. Briefly, the mice were anesthetized using isoflurane inhalation. Subsequently, both common carotid arteries were exposed and isolated from the vagus nerves via a midline incision. A micro-coil with an inner diameter of 0.18 ​mm (Sawane Spring Co. Ltd., Tokyo, Japan) was carefully wound around each common carotid artery. After the procedure, the incision was sutured, and the mice were kept on a heating panel until they regained consciousness. The sham-treated animals underwent the same procedure; however, micro-coils were not introduced around their arteries. Postoperatively, all the mice were returned to their standard cages with unrestricted access to food and water for recovery.

For the anti-RGMa antibody administration experiment, after establishing sham and BCAS mice, isotype control antibody (palivizumab, 10 ​mg/kg) and humanized anti-RGMa antibody (unasnemab/MT-3921, 10 ​mg/kg) were injected intraperitoneally twice a week, starting three days post-surgery. Mice received a total of nine injections; however, in experiments that included behavioral testing, two injections were skipped.

Animals were randomly assigned to experimental groups. Although power calculations to predetermine sample sizes were not conducted, our sample sizes are comparable to those reported in previous studies [22,38]. The number of subjects used in the experiments is reported in the figure legends. Mice that exhibited signs of seriously compromised health during the experiments were excluded.

### Thymidine analog 5-bromo-2-deoxyuridine (BrdU) administration

BrdU (ab142567, Abcam, Cambridge, MA, USA) was diluted in sterile saline and administered via intraperitoneal injection at a dose of 100 ​mg/kg [38,39].

Short-term Proliferation Study (Fig. 3a Left): BrdU integrates into the DNA of dividing cells during the S phase of the cell cycle, which lasts for approximately 8 ​h. Post-injection bioavailability was estimated at 2 ​h. Three weeks postoperatively (in either sham or BCAS group), the mice received two BrdU injections: one at 4 ​h and another 2 ​h before they were sacrificed.

Long-term Cell Survival Study (Fig. 3a Right): Based on reports suggesting that most newborn cells die within the first 7 days after BrdU injection [40], we opted for a two-week survival period for our study. Mice from both the BCAS and sham groups were administered daily BrdU injections from postoperative days 21–25, receiving a total of five injections. They were subsequently sacrificed on postoperative day 35.

Anti-RGMa Antibody Assessment: To determine the effects of the anti-RGMa antibody, all three groups (treated with either isotype control antibody or the anti-RGMa antibody) were administered BrdU daily from postoperative days 7–10, for a total of four injections. All the mice were sacrificed on postoperative day 35 for further analysis.

### CBF measurement by laser speckle flowmetry

CBF was measured as previously reported, with some modifications [37]. Measurements were recorded before BCAS (pre) and 1 and 7 days after BCAS surgery for model validation. Additionally, CBF was recorded 14 days after BCAS surgery in the anti-RGMa antibody administration experiments. Briefly, two days before the first CBF measurement, the mice were anesthetized using isoflurane. A midline incision was made to expose the skull, with the periosteum cleared using cotton swabs. Dental cement was applied to the skull's edge to form a bank, followed by disinfection. The exposed skulls were then sealed with silicone.

For the CBF measurements, mice were anesthetized with 4 % isoflurane and then maintained at 1.5 %. The mice were secured in a stereotaxic frame and placed on a warming pad to ensure a rectal temperature between 36.0 ​°C and 37.5 ​°C. After removing the silicone, phosphate-buffered saline (PBS) was added to the dental cement bank to enhance imaging. We gauged CBF using laser speckle flowmetry with a 780 ​nm laser. Regions of interest were measured in both hemispheres. The data were derived by averaging 250 consecutive raw speckle images and then averaging the CBF values from both the left and right sides. We repeated this process three times and averaged the results to obtain the final CBF value. After the measurement, we resealed the exposed skull with silicone. Mice exhibiting CBF levels over 80 ​% of preoperative values on the first day after surgery were excluded.

### RetroBeads injection

Mice were anesthetized using a mixture of three anesthetic agents. Subsequently, 0.5 ​mL of RetroBeads™ IX Red (Lumafluor Inc.) was injected into the brain using custom-pulled glass pipettes at coordinates of 2.5 ​mm caudal, ±1.5 ​mm lateral, and 1.9 ​mm ventral from the bregma, delivering the substance over 5 ​min at a rate of 100 ​nL/min. Post-surgery, the mice were closely monitored for complications, and their brains were harvested 7 days after the injection.

### Immunohistochemistry

After mice were anesthetized with a mixture of 0.3 ​mg/kg medetomidine hydrochloride (ZENOAQ), 4 ​mg/kg midazolam (Maruishi Pharmaceutical Co., Ltd.), and 5 ​mg/kg butorphanol (Meiji Animal Health Co., Ltd.), they were transcardially perfused with 4 % paraformaldehyde (PFA) in 0.1 ​M phosphate buffer. The brains were obtained following perfusion, post-fixed in 4 % PFA overnight at 4 ​°C, and subsequently transferred into a 30 % sucrose PBS solution at 4 ​°C. The tissues were embedded in Tissue-Tek O.C.T. Compound (Sakura Finetek) and stored at −80 ​°C until use. The brains were cut into 30-μm-thick coronal sections using a cryostat and placed on MAS-coated glass slides (Matsunami). For immunohistochemistry, after washing three times with PBS, the sections were blocked with PBS containing 0.3 % Triton X-100 or 0.05 % Tween 20 and 5 % Normal donkey serum or 5 % Normal goat serum for 1 ​h at room temperature. Subsequently, the sections were incubated with primary antibodies in blocking buffer overnight at 4 ​°C. The primary antibodies used were goat anti- RGMa antibody (1:50; AF2458, R&D), rabbit anti-Neogenin (NEO1) antibody (1:1000; ab190263, abcam), rabbit anti-Doublecortin (DCX) antibody (1:500; 4604, Cell signaling), sheep anti- DCX antibody (1:200; AF10025, R&D), goat anti-Choline Acetyltransferase (ChAT) antibody (1:100; AB114P, Millipore), rat anti-BrdU antibody (1:200; ab6326, abcam), rabbit anti-NeuN antibody (1:500; ab104225, abcam), rabbit anti-Sox2 antibody (1:500; AB5603, Millipore), rat anti-myelin basic protein (MBP) antibody (1:200, NB600-717, NOVUS), rabbit anti-Cleaved caspase-3 antibody (1:1000; 9661, Cell signaling), and Alexa Fluor 647 goat anti-human IgG (1:500; A21445, Invitrogen). For the goat anti-RGMa antibody, rabbit anti-Neogenin antibody, and rat anti-BrdU antibody, we performed antigen retrieval using a citrate buffer (pH 6.0) before the blocking step. The sections were washed three times with PBS and incubated with secondary antibodies in a blocking solution for 1 ​h at room temperature in the dark. The secondary antibodies were as follows: Alexa Fluor 488 donkey anti-rabbit IgG (1:500; A21206, Invitrogen), Alexa Fluor 647 donkey anti-goat IgG (1:500,A21447, Invitrogen), Alexa Fluor 488 goat anti-rabbit IgG (1:500; A11008, Invitrogen), Alexa Fluor647 goat anti-rat IgG (1:500; A21247, Invitrogen), and Alexa Fluor 647 goat anti-rabbit IgG (1:500; A21244, Invitrogen). Nuclei were visualized with DAPI staining, and sections were washed three times with PBS before being mounted in fluorescence mounting medium (Dako).

### Image capture and quantification

All images were acquired using a confocal laser scanning microscope (FV3000, Olympus) according to the manufacturer's software. Six to eight sections ranging from −1.6 ​mm to −2.56 ​mm from the bregma were analyzed for hippocampal quantification for each mouse, while four sections between +1.1 ​mm and +0.62 ​mm from the bregma were used for the quantification of the corpus callosum and the medial septum (MS) and the diagonal band of Broca (DBB).

Quantitative analysis was performed using the ImageJ software (v2.1.0/1.53c), with data quantification performed by researchers blinded to the experimental conditions. The fluorescence intensities of RGMa within the dentate gyrus and corpus callosum were corrected. The intensity of RGMa in the NeuN-positive area within the dentate gyrus was also assessed. Using the ImageJ cell counter plugin, we quantified the cells positive for DCX, NeuN, DCX/BrdU, and NeuN/BrdU and normalized them to the granular cell layer (GCL) and SGZ area. Sox2-, BrdU-, cleaved caspase-3-, and cleaved caspase-3/DCX-positive cells were counted and normalized to the SGZ. ChAT-positive cells were quantified and normalized to the MS/DBB area. Threshold analysis in ImageJ was employed to evaluate the MBP-positive region in the corpus callosum and the ChAT-positive region in the dentate gyrus. To ensure staining specificity, we performed concentration-matched isotype control IgG staining.

### Western blotting

Mice were deeply anesthetized using a mixture of three anesthetic agents and transcardially perfused with ice-cold 0.1 ​M phosphate buffer 5 weeks after the BCAS or sham operation. Hippocampi were obtained following perfusion. Tissues were lysed in a lysis buffer containing RIPA Lysis Buffer (20–188, Millipore) and Protease/Phosphatase Inhibitor Cocktail (5872, Cell signaling). Each sample was heated in sample buffer (161–0737, Bio-Rad) and 2-Mercaptoethanol at 95 ​°C for 5 ​min, resolved by sodium dodecyl sulfate-polyacrylamide gel electrophoresis, and transferred to PVDF membranes (1704156, Bio-Rad). Membranes were blocked with PBS containing 0.05 % Tween 20 and 5 % skim milk for 1 ​h, then incubated with goat anti- RGMa antibody (1:500; AF2458, R&D), goat anti-Neogenin (NEO1) antibody (1:1000, AF1079, R&D), and rabbit anti-β-actin (1:1000; 4970, Cell Signaling) overnight at 4 ​°C. The membranes were then incubated with horseradish peroxidase-conjugated secondary antibodies against goat IgG (1:10000; sc-2020; Santa Cruz Biotechnology) and rabbit IgG (1:10000; 7074S; Cell Signaling) for 1 ​h at room temperature. Detection was performed using an enhanced chemiluminescence substrate (34095 and A38554; Thermo Fisher Scientific). All pictures were obtained using a ChemiDoc MP Imaging System (Bio-Rad). The intensity of the protein bands was quantified using the ImageJ software (v2.1.0/1.53c). β-Actin bands were used for normalization.

### Behavior test

Prior to the behavioral test, the mice were acclimated to human interaction to minimize stress during the experiments. They were handled daily for a minimum of 3 ​min over three consecutive days. Additionally, to acclimate them to the test environment, they were placed in the experimental room 1 ​h before each session. Behavioral tests were conducted five weeks post-surgery.

### Barnes maze test

We used the Barnes maze, a tool specifically designed to evaluate spatial learning and memory, as previously described, with some modifications [41]. The maze, manufactured by SHINFACTORY, consisted of a white circular platform measuring 91 ​cm in diameter and 112 ​cm in height. Twenty circular holes were evenly spaced along the outer edge of the platform, with one of them linked to a dark escape box. Overhead room lights brightly illuminated the maze, and visual cues on the surrounding walls helped guide the animals. The maze and escape boxes were cleaned with 70 % ethanol to eliminate any olfactory cues during each trial.

A habituation session was conducted one day before the acquisition session to familiarize the mice with the maze. During this session, each mouse was placed in the center of the maze and led to an escape chamber, where they stayed for 30 ​s. This habituation routine was repeated five times before the mice were returned to their home cages.

For the acquisition session, the escape box was consistently placed at the same location as it was during the habituation phase, ensuring its fixed position relative to the room's spatial cues. Over four consecutive days, the mice were individually placed at the center of the maze and allowed to explore. Each trial ended when the mouse entered the escape box or after 5 ​min had elapsed. Mice that could not enter the escape box within this 5-min timeframe were guided to it and allowed a 1-min stay before removal. The time and distance taken to locate the escape hole were measured.

One day after the final acquisition trials, a 90-s probe trial was conducted to assess the retention of spatial memory. During this trial, the escape box was removed from the maze, and the mice were allowed to explore freely for 90 ​s. The time spent by each mouse in the quadrant containing escape holes was recorded.

Throughout all sessions, the animals' movements and behaviors were captured and analyzed using the SMART Video-Tracking System.

### Novel object recognition test (NORT)

NORT was performed as previously described, with some modifications [42]. The test comprised three stages: habituation, familiarization, and test sessions. On the first day, the mice underwent a 15-min habituation phase, during which they were placed in a square experimental box with specific dimensions (40 ​cm ​× ​40 ​cm ​× ​40 ​cm) devoid of objects. The following day, the mice spent 5 ​min in the same empty box before being returned to their home cages. Subsequently, two identical objects were placed in the box and positioned 10 ​cm away from the walls. The mice were reintroduced into the box to familiarize themselves with the objects for 10 ​min. After a 12-h interval, for the test session, one of the previously introduced objects and a new object were positioned in the same locations. The mice were allowed to explore the area for 10 ​min. An overhead camera captured their behavior, and exploration time for each object was measured manually.

The discrimination index (DI) was calculated using the following formula: DI = (duration: novel object – duration: familiar object)/(duration: novel object ​+ ​duration: familiar object). Exploration was defined as the direct interaction or observation of the object within a 2-cm distance. However, climbing atop an object was not considered as exploration. The box and objects were cleaned with ethanol to avoid olfactory cues between trials.

### Quantification and statistical analysis

All data are presented as means ​± ​standard error of the mean (SEM), with statistical significance set at *p* ​< ​0.05. The unpaired two-sided Student's *t*-test was used for comparisons between two groups, and one-way or two-way analysis of variance (ANOVA) with Tukey's multiple comparison test was used for multiple groups. Statistical analyses were conducted using GraphPad Prism 8 (GraphPad Software).

## Results

### RGMa is increased in the hippocampal dentate gyrus in the BCAS model

**Fig. 1:**
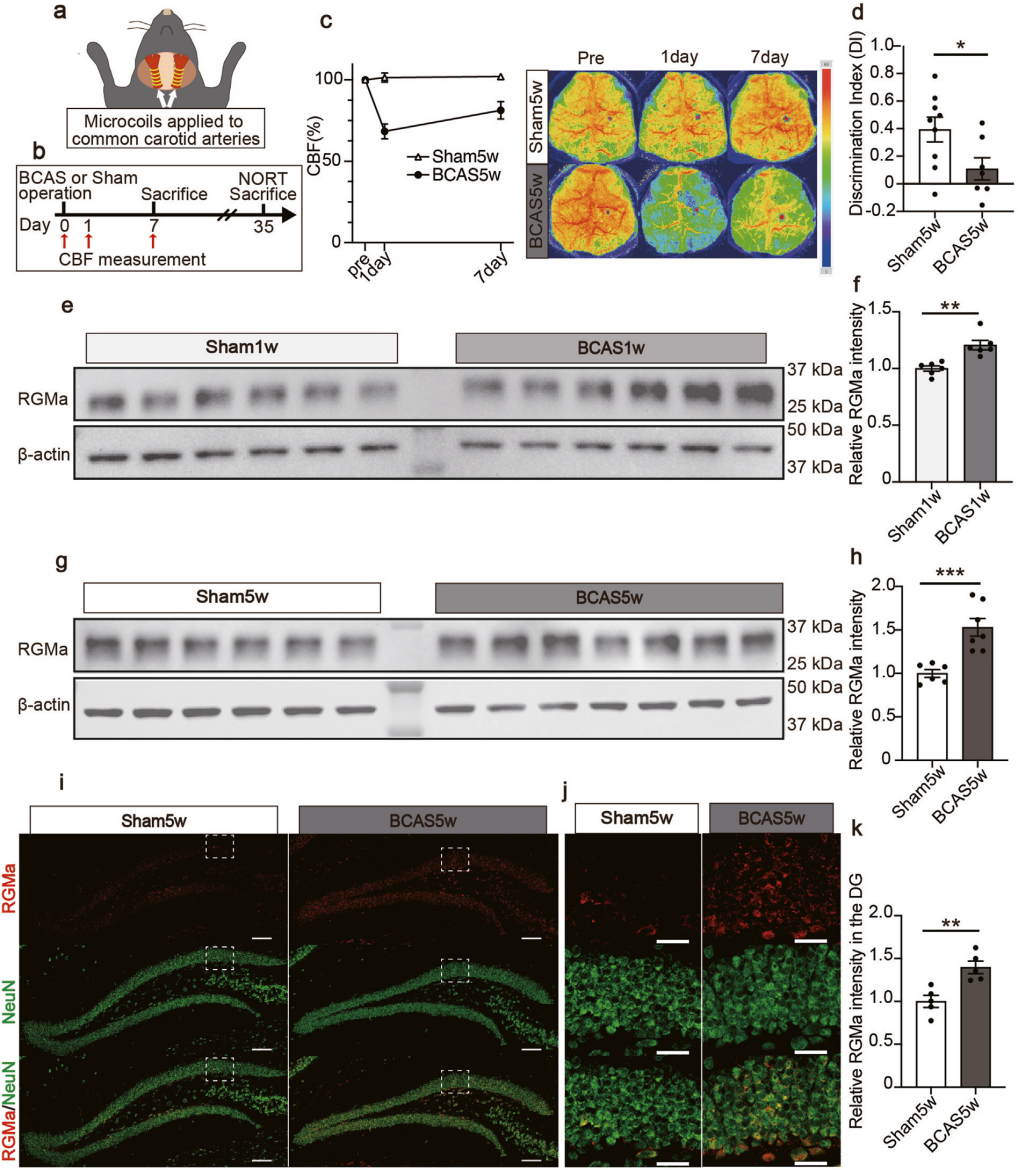
RGMa is increased in the hippocampal dentate gyrus in the BCAS model. **a** Schema of the BCAS surgery. A micro-coil was applied to each common carotid artery. **b** Experimental timeline detailing CBF measurements, NORT, and tissue collection. Initially, CBF measurements were taken prior to the BCAS or Sham surgery, as well as 1 and 7 days post-surgery. On the 7th postoperative day, or after performing NORT on the 35th postoperative day, the mice were sacrificed for immunohistochemistry and western blotting analyses. **c** Temporal changes in CBF relative to baseline after BCAS or sham surgery. The right panel is representative laser speckle flowmetry images taken before surgery (pre) and 1 and 7 days postoperatively. **d** Discrimination index for exploring a novel object five weeks post-surgery during the NORT. Mean ​± ​SEM; n ​= ​9 (sham5w), n ​= ​7 (BCAS5w); unpaired Student's t-test: ∗*p* ​< ​0.05. **e** Western blot for RGMa in the hippocampus 1 week post-surgery. **f** Relative Western blot density quantification of RGMa in the hippocampus 1 week post-surgery. Mean ​± ​SEM; n ​= ​6 (sham1w), n ​= ​6 (BCAS1w); unpaired Student's t-test: ∗∗*p* ​< ​0.01. **g** Western blot for RGMa in the hippocampus 5 weeks post-surgery. **h** Relative Western blot density quantification of RGMa in the hippocampus 5 weeks post-surgery. Mean ​± ​SEM; n ​= ​6 (sham5w), n ​= ​7 (BCAS5w); unpaired Student's t-test: ∗∗∗*p* ​< ​0.001. **i** Representative images of RGMa and NeuN staining in the dentate gyrus 5 weeks post-surgery. Scale bars: 100 ​μm. **j** Magnified view of the boxed area in (**g**). Scale bars: 30 ​μm. **k** The graph shows the relative RGMa intensity in the DG at 5 weeks post-surgery. n ​= ​5 (sham5w), n ​= ​5 (BCAS5w). Mean ​± ​SEM; n ​= ​5 (sham5w), n ​= ​5 (BCAS5w); unpaired Student's t-test: ∗∗*p* ​< ​0.01. BCAS, bilateral common carotid artery stenosis; CBF, cerebral blood flow; NORT, novel object recognition test; RGMa, Repulsive Guidance Molecule A; SEM, standard error of means; DG, dentate gyrus.

To determine whether RGMa was involved in the pathology of VaD, we generated a BCAS model in mice. The BCAS model was established by placing micro-coils bilaterally around the common carotid arteries (Fig. 1a and b) [8,43,44]. First, to validate our model, we measured CBF. CBF was monitored preoperatively and on the first and seventh postoperative days. Consistent with previous studies [43,45,46], our BCAS mice exhibited a decrease in CBF to 68.43 % (mean ​± ​SEM) of preoperative values on the first day after surgery, which recovered to 81.29 % (mean ​± ​SEM) by the seventh day post-surgery (Fig. 1c). Next, we performed behavioral tests to confirm whether BCAS-treated mice exhibited cognitive impairment. Given that BCAS mice typically exhibit cognitive deficits at approximately 4 weeks post-surgery [44,47,48], we assessed their cognitive function using the NORT at 5 weeks post-surgery and confirmed that the BCAS mice had impaired performance in recognizing novel objects, as expected (Fig. 1d).

Next, we aimed to compare the RGMa expression in the hippocampus between the sham and BCAS groups. Western blotting data revealed a pronounced increase in RGMa expression within the hippocampus of the BCAS group at 1 and 5 weeks post-surgery. Using Western blotting, we observed a significant increase in the 33-kDa C-terminal-processed form of RGMa in the hippocampus of post-BCAS surgery mice compared to sham mice (Fig. 1e–h). There was no significant difference in NEO1 expression at one week post-surgery between the two groups (Figs. S1a and b).

Furthermore, immunohistochemical analysis evaluated RGMa expression in the dentate gyrus, which is the site of neurogenesis. RGMa-immunoreactive intensities were significantly elevated in the dentate gyrus of the BCAS model mice (Fig. 1i–k). Notably, in both groups, the primary location of RGMa expression was the GCL, where NeuN-positive mature granule neurons were present (Fig. 1j). In contrast, RGMa was not upregulated in the corpus callosum, which is one of the primary sites affected in individuals with VaD (Figs. S2a and b) [5,49].

In contrast, the receptor for RGMa, neogenin, was expressed in DCX-positive immature neurons within the SGZ, which is consistent with a previous study (Fig. S1c) [36]. The ratio of NEO1+ DCX ​+ ​cells among DCX ​+ ​cells in the dentate gyrus of control mice was 39.5 ​%.

### Hippocampal neurogenesis is impaired in BCAS mice

**Fig. 2:**
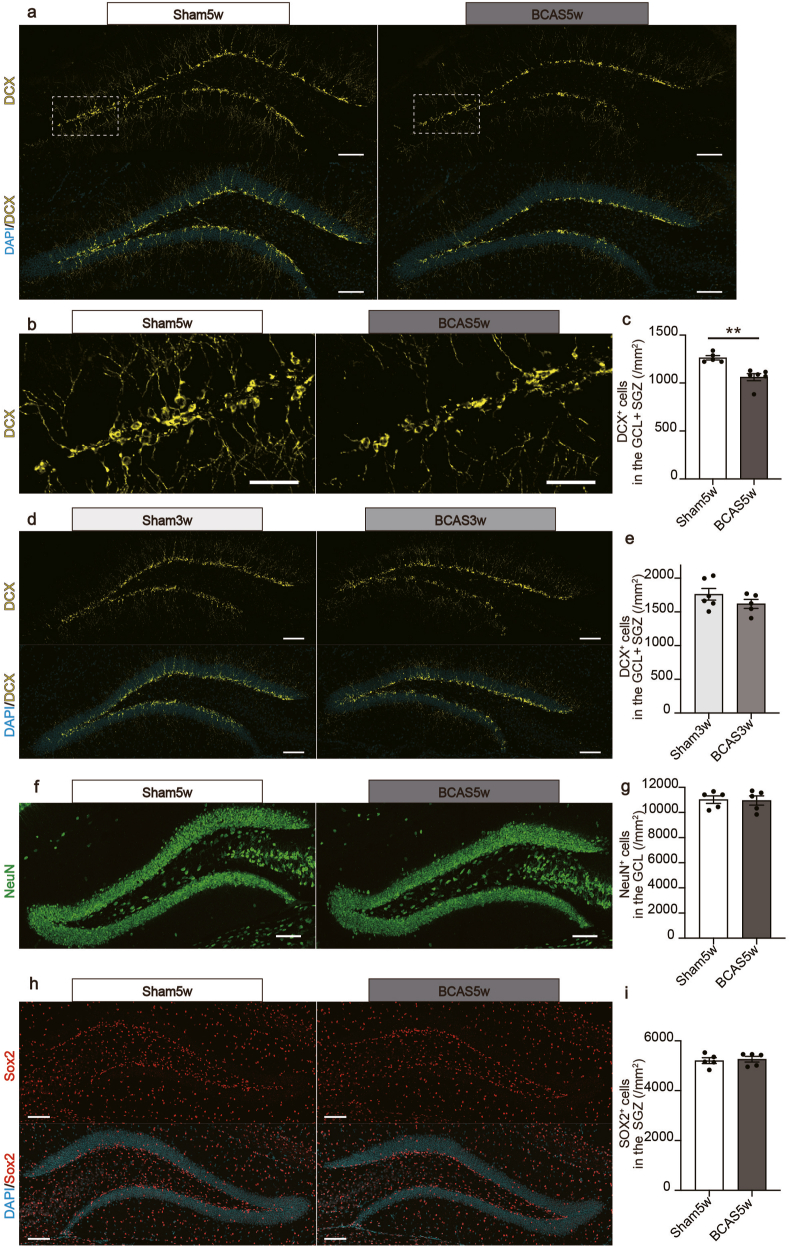
Hippocampal neurogenesis is impaired in BCAS mice at five weeks post-surgery. **a** Representative images of DCX staining in the dentate gyrus at 5 weeks post-surgery. Scale bars: 100 ​μm. **b** Magnified views of the boxed area in (**j**). Scale bars: 50 ​μm. **c** Quantitative data showing the number of DCX-positive cells in the GCL ​+ ​SGZ area at 5 weeks post-surgery. Mean ​± ​SEM; n ​= ​5 (sham5w), n ​= ​5 (BCAS5w); unpaired Student's t-test: ∗∗*p* ​< ​0.01. **d** Representative images of DCX staining in the dentate gyrus at 3 weeks post-surgery. Scale bars: 100 ​μm. **e** Quantitative data displaying the number of DCX-positive cells in the GCL ​+ ​SGZ area at 3 weeks post-surgery. Mean ​± ​SEM; n ​= ​6 (sham3w), n ​= ​5 (BCAS3w); unpaired Student's t-test. **f** Representative images of NeuN staining in the dentate gyrus at 5 weeks post-surgery. Scale bars: 100 ​μm **g** The graph on the right presents quantitative data displaying the number of NeuN-positive cells in the GCL area at 5 weeks post-surgery. Mean ​± ​SEM; n ​= ​5 (sham5w), n ​= ​5 (BCAS5w); unpaired Student's t-test. **h** Representative images of Sox2 staining in the dentate gyrus at 5 weeks post-surgery. Scale bars: 100 ​μm. **i** Quantitative data displaying the number of Sox2-positive cells in the SGZ area at 5 weeks post-surgery. Mean ​± ​SEM; n ​= ​5 (sham5w), n ​= ​5 (BCAS5w); unpaired Student's t-test. BCAS, bilateral common carotid artery stenosis; DCX, doublecortin; GCL, granular cell layer; SEM, standard error of means; SGZ, subgranular zone.

To test the temporal correlation between impaired neurogenesis and elevated RGMa expression in the BCAS model, we compared the number of immature DCX-positive neurons between the BCAS and sham groups using immunohistochemistry at 5 weeks post-surgery. BCAS mice had fewer immature DCX-positive neurons in the GCL and SGZ than sham mice (Fig. 2a–c). However, by the third postoperative week, both groups had similar DCX-positive cell populations (Fig. 2d and e).

**Fig. 3:**
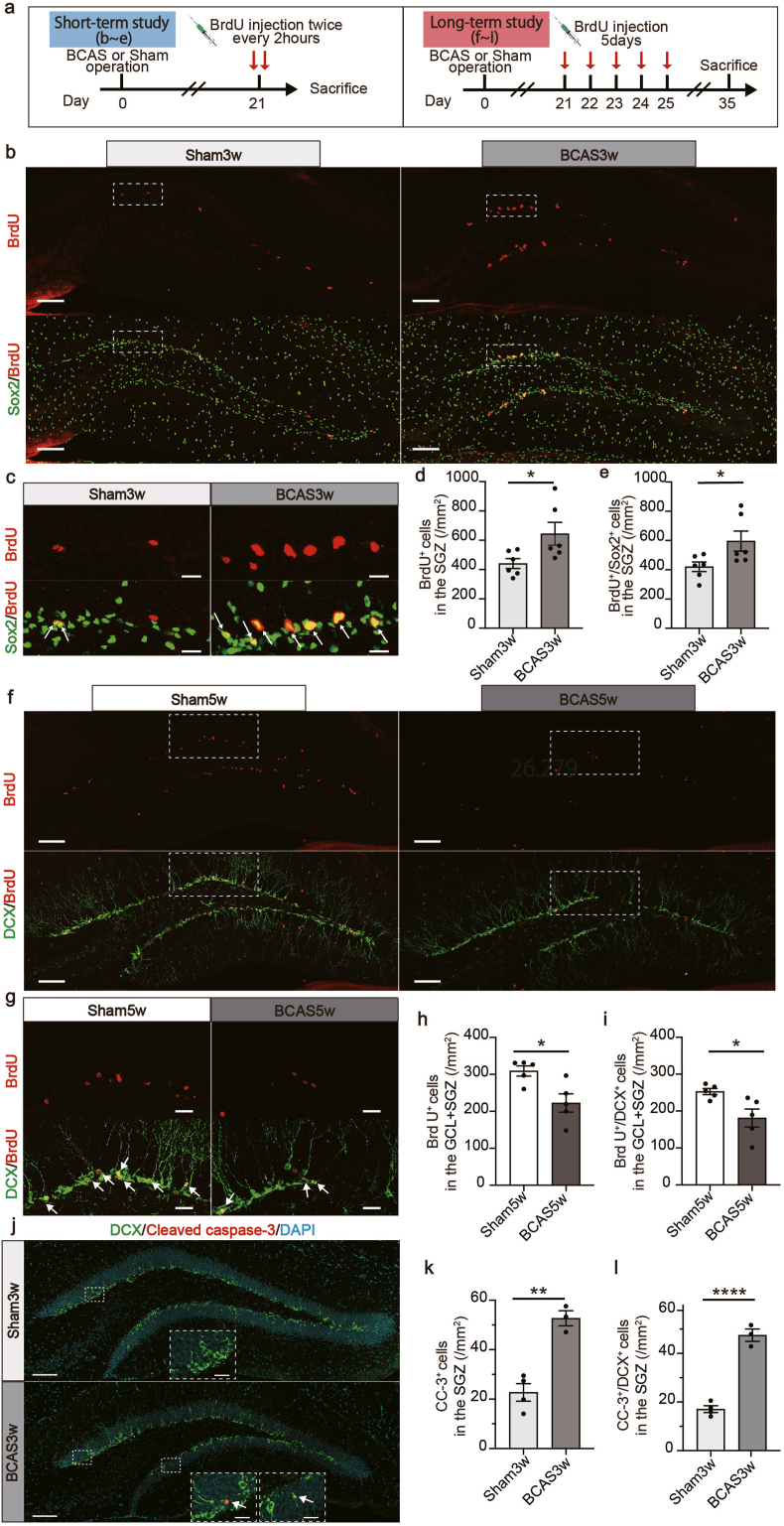
Decreased DCX-positive cell population resulted from disrupted differentiation rather than proliferation. **a** Experimental timeline detailing the design to assess short-term proliferation and long-term survival of new-born neurons in BCAS mice. Left: For short-term proliferation, 3 weeks after surgery, both BCAS and sham mice were administered two injections of the mitotic marker bromodeoxyuridine (BrdU) at 2-h intervals before being sacrificed. Right: For long-term cell survival, BCAS and sham mice were given a single daily BrdU injection from postoperative days 21 through 25, making a total of five injections. Mice were then sacrificed on day 35. **b** Representative images of BrdU and Sox2 staining in the dentate gyrus at 3 weeks post-surgery. Scale bars: 100 ​μm. **c** Magnified views of the indicated boxed area in (**b**) with scale bar set at 20 ​μm. White arrows indicate BrdU and Sox2-positive cells. **d** Quantitative data displaying the number of BrdU-positive cells in the SGZ area at 3 weeks post-surgery. Mean ​± ​SEM; n ​= ​6 (sham3w), n ​= ​6 (BCAS3w); unpaired Student's t-test: ∗*p* ​< ​0.05. **e** Quantitative data displaying the number of BrdU and Sox2-positive cells in the SGZ area at 3 weeks post-surgery. Mean ​± ​SEM; n ​= ​6 (sham3w), n ​= ​6 (BCAS3w); unpaired Student's t-test: ∗*p* ​< ​0.05. **f** Representative images of BrdU and DCX staining in the dentate gyrus at 5 weeks post-surgery. Scale bars: 100 ​μm. **g** Magnified views of the indicated boxed area in (**f**) with scale bar set at 50 ​μm. **h** Quantitative data displaying the number of BrdU-positive cells in the GCL ​+ ​SGZ area at 5 weeks post-surgery. Mean ​± ​SEM; n ​= ​5 (sham5w), n ​= ​5 (BCAS5w); unpaired Student's t-test: ∗*p* ​< ​0.05. **i** Quantitative data displaying the number of BrdU and DCX-positive cells in the GCL ​+ ​SGZ area at 5 weeks post-surgery. Mean ​± ​SEM; n ​= ​5 (sham5w), n ​= ​5 (BCAS5w); unpaired Student's t-test: ∗*p* ​< ​0.05. **j** Representative images of cleaved caspase-3 and DCX staining in the dentate gyrus at 3 weeks post-surgery. Scale bars: 100 ​μm. The lower insets display magnified views of the indicated boxed areas, with scale bars set at 10 ​μm. White arrows indicate cells that are positive for both cleaved caspase-3 and DCX. **k** Quantitative data displaying the number of cleaved caspase-3-positive cells in the SGZ area at 3 weeks post-surgery. Mean ​± ​SEM; n ​= ​3 (sham3w), n ​= ​3 (BCAS3w); unpaired Student's t-test: ∗∗*p* ​< ​0.01. **l** Quantitative data displaying the number of cleaved caspase-3 and DCX-positive cells in the SGZ area at 3 weeks post-surgery. Mean ​± ​SEM; n ​= ​3 (sham3w), n ​= ​3 (BCAS3w); unpaired Student's t-test: ∗∗∗∗*p* ​< ​0.0001. BCAS, bilateral common carotid artery stenosis; DCX, doublecortin; GCL, granular cell layer; SEM, standard error of means; SGZ, subgranular zone.

Subsequent immunohistochemical analyses using NeuN for mature neurons and Sox2 for neural stem cells revealed no significant variations in NeuN-positive cells in the GCL (Fig. 2f and g) or in Sox2-positive cells in the SGZ (Fig. 2h and i). In BCAS mice, our results indicated that DCX-positive cells, which are involved in the process of differentiation, were more vulnerable to damage from chronic hypoperfusion than other neuronal cells. This reduction became apparent between three and five weeks postoperatively, underscoring the BCAS model's progressive impact.

### Decreased DCX-positive cell population resulted from disrupted differentiation rather than proliferation

To determine whether impaired proliferation or differentiation in the biphasic process of neurogenesis accounted for the reduction of DCX-positive cells observed five weeks after BCAS surgery, we administered BrdU three weeks post-surgery (Fig. 3a). BrdU, a synthetic thymidine analog, is incorporated into the DNA of dividing cells [39]. In our short-term proliferation study, mice received two BrdU injections at intervals of 4 and 2 ​h before euthanasia. For the long-term differentiation study, we administered daily BrdU injections from postoperative days 21–25, totaling five injections. Brain samples were collected 2 weeks following the BrdU injection (Fig. 3a). In the short-term proliferation study, BCAS mice exhibited a significant increase in the number of BrdU- and BrdU/Sox2-positive cells compared to the sham group (Fig. 3b–e). Conversely, the long-term cell survival study revealed a decrease in BrdU-positive cells and newly generated DCX-positive neurons labeled with BrdU in the BCAS group that persisted for 2 weeks compared to the sham group (Fig. 3f–i). BCAS mice also exhibited a greater number of cleaved caspase-3-positive cells and cleaved caspase-3/DCX double-positive cells in the SGZ than sham mice at 3 weeks post-surgery (Fig. 3j–l). These results suggest that a decrease in cell survival could contribute to the decline in the DCX-positive cell population 5 weeks after BCAS surgery. Furthermore, to counteract this cell loss, cell proliferation appears to be upregulated.

### Anti-RGMa antibody treatment attenuates BCAS-induced impairment of neurogenesis

**Fig. 4:**
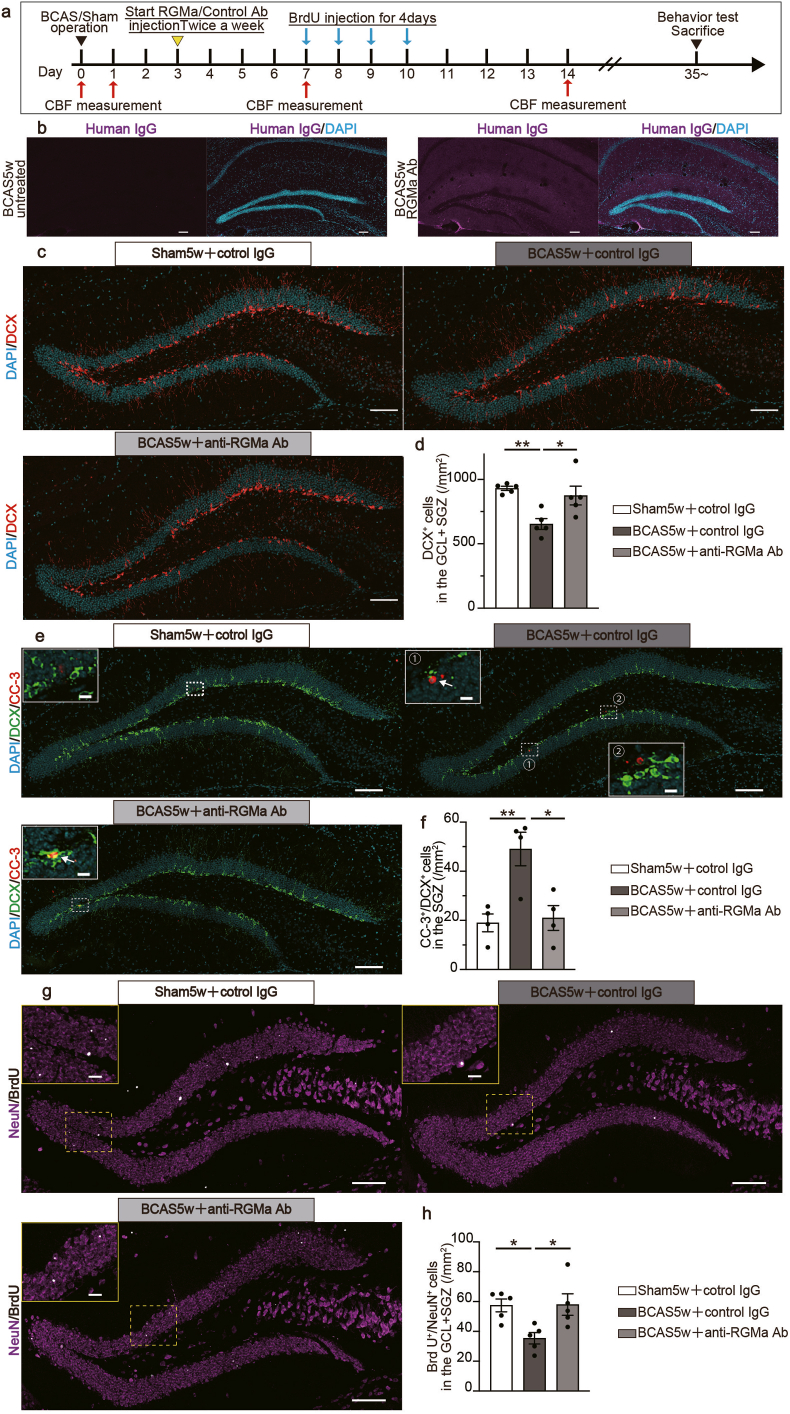
Anti-RGMa antibody ameliorates the decrease in DCX-positive cells and the survival rate of newly born neurons in the BCAS model. **a** Experimental timeline for the anti-RGMa antibody administration study. Mice underwent CBF measurement prior to BCAS or sham surgery (pre) and again at 1, 7, and 14 days post-surgery. A humanized RGMa-mAb or an IgG isotype control antibody was subsequently administered intraperitoneally twice weekly, for either 7 or 9 times. To label dividing cells, a BrdU injection was given daily from postoperative days 7 through 10, totaling four injections. After undergoing behavior tests around postoperative day 35, the mice were euthanized. **b** Representative images of human IgG staining in the dentate gyrus with or without humanized anti-RGMa antibody injected BCAS mouse. Scale bars: 100 ​μm. **c** Representative images of DCX staining in the dentate gyrus at 5 weeks post-surgery. Scale bars: 100 ​μm. **d** Quantitative data displaying the number of DCX-positive cells in the GCL ​+ ​SGZ area at 5 weeks post-surgery. n ​= ​5 (sham5w ​+ ​control IgG), n ​= ​5 (BCAS5w ​+ ​control IgG), n ​= ​5 (BCAS5w ​+ ​anti-RGMa Ab); one-way ANOVA with Tukey's multiple comparisons test: sham5w ​+ ​control IgG vs BCAS5w ​+ ​control IgG: ∗∗*p* ​< ​0.01, BCAS5w ​+ ​control IgG vs BCAS5w ​+ ​anti-RGMa Ab: ∗*p* ​< ​0.05. **e** Representative images of cleaved caspase-3 and DCX staining in the dentate gyrus at 5 weeks post-surgery. Scale bars: 100 ​μm. **f** Quantitative data displaying the number of cleaved caspase-3- and DCX -positive cells in the GCL ​+ ​SGZ area at 5 weeks post-surgery. n ​= ​4 (sham5w ​+ ​control IgG), n ​= ​4 (BCAS5w ​+ ​control IgG), n ​= ​4 (BCAS5w ​+ ​anti-RGMa Ab); one-way ANOVA with Tukey's multiple comparisons test: sham5w ​+ ​control IgG vs BCAS5w ​+ ​control IgG: ∗∗*p* ​< ​0.01, BCAS5w ​+ ​control IgG vs BCAS5w ​+ ​anti-RGMa Ab: ∗*p* ​< ​0.05. **g** Representative images of BrdU and NeuN staining in the DG 5 weeks post-surgery. Scale bars: 100 ​μm. The upper left insert provides a magnified view of the indicated boxed area, with a scale bar of 20 ​μm ​ **h** Quantitative data showing the number of BrdU- and NeuN-positive cells in the GCL ​+ ​SGZ area at 5 weeks post-surgery. Mean ​± ​SEM; n ​= ​5 (sham5w ​+ ​control IgG), n ​= ​5 (BCAS5w ​+ ​control IgG), n ​= ​5 (BCAS5w ​+ ​anti-RGMa Ab); one-way ANOVA with Tukey's multiple comparisons test: sham5w ​+ ​control IgG vs BCAS5w ​+ ​control IgG: ∗*p* ​< ​0.05, BCAS5w ​+ ​control IgG vs BCAS5w ​+ ​anti-RGMa Ab: ∗*p* ​< ​0.05. BCAS, bilateral common carotid artery stenosis; CBF, cerebral blood flow; DCX, doublecortin; GCL, granular cell layer; SEM, standard error of means; SGZ, subgranular zone; DG, dentate gyrus.

In the next step, we assessed the potential therapeutic impact of the anti-RGMa antibody on BCAS-induced impairment of neurogenesis. Starting from the third postoperative day, we administered either anti-RGMa antibody or control IgG intraperitoneally twice a week. On the seventh day, to elucidate the antibody's role in neurogenic differentiation, we administered BrdU intraperitoneally daily for four consecutive days (Fig. 4a). First, we investigated whether the anti-RGMa antibody enters the hippocampus of BCAS mice by performing immunohistochemistry for human IgG. In BCAS mice treated with the anti-RGMa antibody, we observed discernible signals in the hippocampus, whereas no signals were detected in untreated BCAS mice (Fig. 4b). Subsequently, we investigated whether CBF was affected by anti-RGMa antibody treatment using laser speckle flowmetry. We found no discernible differences in CBF between the BCAS-control IgG and BCAS-*anti*-RGMa antibody-treated groups (Figs. S3a and b).

Next, we analyzed the number of DCX-positive cells. We found that anti-RGMa antibody treatment effectively mitigated the reduction in DCX-positive cells induced by BCAS surgery (Fig. 4c and d). Additionally, anti-RGMa antibody treatment reduced the number of cleaved caspase-3/DCX double-positive cells induced by BCAS surgery (Fig. 4e and f). In the control IgG-treated BCAS mice, the number of BrdU ​+ ​NeuN ​+ ​cells in the dentate gyrus was lower than that in the control IgG-treated sham group. This reduction was significantly reversed by anti-RGMa antibody treatment (Fig. 4g and h), indicating its potential role in promoting the survival and differentiation of newborn cells. Notably, in the white matter lesion, which is one of the primary features in patients with VaD [5,49], the anti-RGMa antibody did not significantly ameliorate the condition caused by BCAS surgery (Figs. S2c and d).

### Anti-RGMa antibody treatment attenuates cognitive impairment

**Fig. 5:**
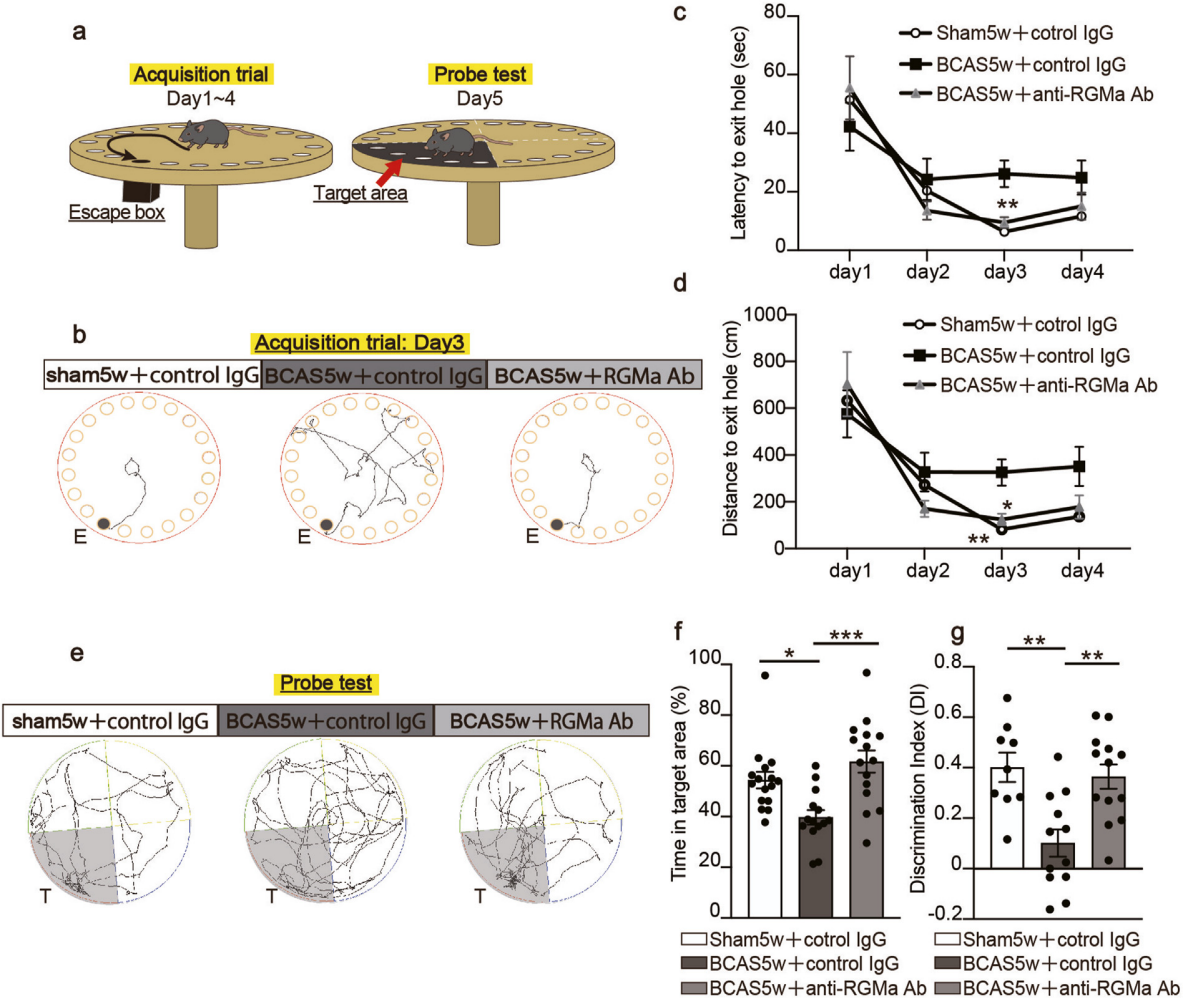
Anti-RGMa antibody alleviates cognitive impairment in the BCAS mice. **a** Schema illustrating the Barnes maze test setup. The Barnes maze evaluation spans 5 days: acquisition trials to acquaint participants with the escape hole's location are conducted from days 1–4 (depicted in the left), and these lead up to a probe test on day 5 (shown on the right). **b** Representative tracking plots from the third day of the Barnes maze training trial. 'E' indicates an exit hole. **c** Analysis of the latency time used by the mice to find the escape box in the acquisition trials. Mean ​± ​SEM; n ​= ​13 (sham5w ​+ ​control IgG), n ​= ​12 (BCAS5w ​+ ​control IgG), n ​= ​9 (BCAS5w ​+ ​anti-RGMa Ab); two-way ANOVA with Tukey's multiple comparisons test: on day 3: sham5w ​+ ​control IgG vs BCAS5w ​+ ​control IgG: ∗∗*p* ​< ​0.01, BCAS5w ​+ ​control IgG vs BCAS5w ​+ ​anti-RGMa Ab: ∗∗*p* ​< ​0.01. **d** Evaluation of the distance traveled by the mice to reach the escape box during the acquisition trials. Mean ​± ​SEM; n ​= ​13 (sham5w ​+ ​control IgG), n ​= ​12 (BCAS5w ​+ ​control IgG), n ​= ​9 (BCAS5w ​+ ​anti-RGMa Ab); two-way ANOVA with Tukey's multiple comparisons test: on day 3: sham5w ​+ ​control IgG vs BCAS5w ​+ ​control IgG: ∗∗P ​< ​0.01, BCAS5w ​+ ​control IgG vs BCAS5w ​+ ​anti-RGMa Ab: ∗*p* ​< ​0.05. **e** Representative tracking plots of the Barnes maze probe test. The gray areas on the platform represent the target area. **f** Analysis of the percentage of time each mouse spent in the target area. Mean ​± ​SEM; n ​= ​13 (sham5w ​+ ​control IgG), n ​= ​12 (BCAS5w ​+ ​control IgG), n ​= ​9 (BCAS5w ​+ ​anti-RGMa Ab); one-way ANOVA with Tukey's multiple comparisons test: sham5w ​+ ​control IgG vs BCAS5w ​+ ​control IgG: ∗*p* ​< ​0.05, BCAS5w ​+ ​control IgG vs BCAS5w ​+ ​anti-RGMa Ab: ∗∗∗*p* ​< ​0.001. **g** Quantitative analysis of the discrimination index in the NORT. Mean ​± ​SEM; n ​= ​13 (sham5w ​+ ​control IgG), n ​= ​12 (BCAS5w ​+ ​control IgG), n ​= ​9 (BCAS5w ​+ ​anti-RGMa Ab); one-way ANOVA with Tukey's multiple comparisons test: sham5w ​+ ​control IgG vs BCAS5w ​+ ​control IgG: ∗∗*p* ​< ​0.01, BCAS5w ​+ ​control IgG vs BCAS5w ​+ ​anti-RGMa Ab: ∗∗*p* ​< ​0.01. BCAS, bilateral common carotid artery stenosis; NORT, novel object recognition test; RGMa, Repulsive Guidance Molecule A, SEM, standard error of means.

To explore the therapeutic effect of the anti-RGMa antibody on cognitive impairment, we initiated a series of behavioral tests starting 5 weeks post-surgery. We initially assessed spatial learning and memory in the three groups using the Barnes maze test (Fig. 5a). Throughout the acquisition trials, all groups exhibited a consistent reduction in both latency times and distances traveled to locate the escape box. This trend indicates that the mice relied on their learned spatial memory to search for the exit hole rather than finding it through random exploration. However, on the third day, the BCAS-control IgG-treated mice exhibited significantly prolonged latency times and distances compared to the sham-control IgG group, suggesting an impairment in spatial learning among the BCAS-control IgG-treated mice. In contrast, BCAS mice that received anti-RGMa antibody treatment showed latency times and distances similar to those of the sham control group, indicating no noticeable impairment in spatial learning (Fig. 5b–d). Subsequently, we conducted a probe test by removing the escape box to ascertain the mice's memory of the target goal's location. The BCAS-control IgG-treated mice spent a significantly shorter time in the target area than the sham-control group, suggesting that the BCAS-control IgG-treated mice experienced difficulties in recalling the location of the exit hole. In contrast, in BCAS mice receiving anti-RGMa antibody treatment, the time spent in the target area was comparable to that in the sham control group (Fig. 5e and f).

To further assess cognitive performance, we employed NORT. The DI, which indicates preference for the novel object, was significantly decreased in BCAS-control IgG-treated mice. Notably, this decrease was markedly ameliorated in mice treated with BCAS-*anti*-RGMa antibodies (Fig. 5g). These findings highlight the potential therapeutic benefits of anti-RGMa antibodies in treating cognitive impairment.

### Anti-RGMa antibody treatment attenuates BCAS-induced loss of cholinergic fibers

**Fig. 6:**
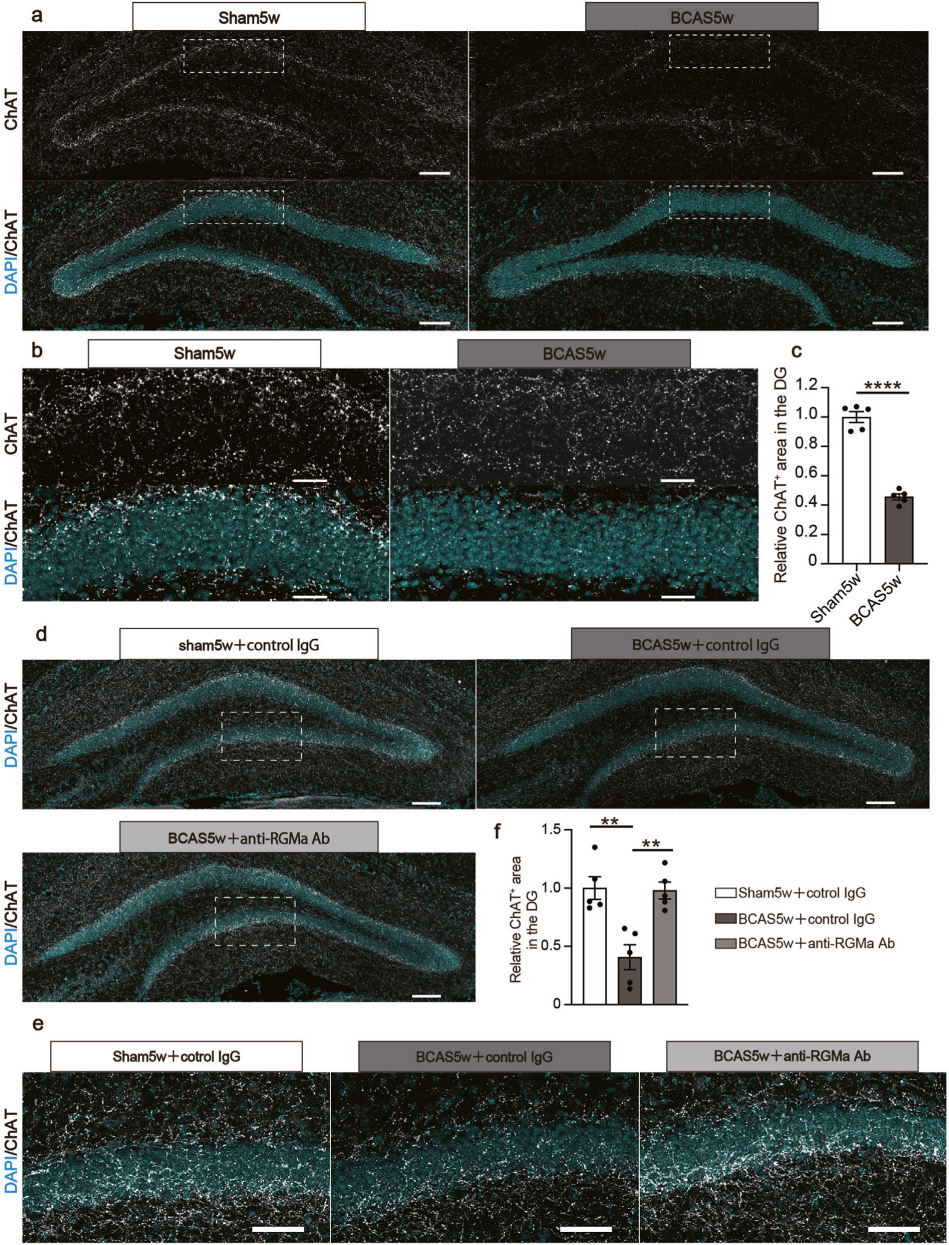
Cholinergic innervation is disturbed in the hippocampal dentate gyrus in the BCAS model, and the anti-RGMa antibody mitigates these cholinergic innervation deficits in BCAS mice. **a** Representative images of ChAT staining in the DG at 5 weeks post-surgery. Scale bars: 100 ​μm. **b** Magnified views of the indicated boxed area in (**a**) with scale bar set at 50 ​μm. **c** Quantitative data displaying the relative ChAT-positive area in the dentate gyrus at 5 weeks post-surgery. Mean ​± ​SEM; n ​= ​5 (sham5w), n ​= ​5 (BCAS5w); unpaired Student's t-test: ∗∗∗∗*p* ​< ​0.0001. **d** Representative images of ChAT staining in the dentate gyrus at 5 weeks post-surgery. Scale bars: 100 ​μm. **e** Magnified views of the indicated boxed area from (**a**). Scale bars: 50 ​μm. **f** Quantitative analysis showing the relative ChAT-positive area in the dentate gyrus 5 weeks post-surgery. Mean ​± ​SEM; n ​= ​5 (sham5w ​+ ​control IgG), n ​= ​5 (BCAS5w ​+ ​control IgG), n ​= ​5 (BCAS5w ​+ ​anti-RGMa Ab); one-way ANOVA with Tukey's multiple comparisons test: ∗∗*p* ​< ​0.01. BCAS, bilateral common carotid artery stenosis; ChAT, choline acetyltransferase; RGMa, Repulsive Guidance Molecule A, SEM, standard error of means; DG, dentate gyrus.

Hippocampus-dependent cognitive functions are modulated by various neurotransmitters, especially acetylcholine [50]. Patients with VaD exhibit reduced levels of acetylcholine in the cerebrospinal fluid and a decline in cholinergic markers in the brain [[51], [52], [53]], which is considered to be involved in the pathophysiology of cognitive impairment. We assessed whether the cholinergic innervation within the dentate gyrus is affected by BCAS using immunohistochemical analysis. Consequently, the density of ChAT-positive fibers was significantly lower in the BCAS group than in the sham group at 5 weeks post-surgery (Fig. 6a–c). However, the total count of cholinergic neurons in the MS and DBB—regions where hippocampus-projecting cholinergic neurons are predominantly located—remained unchanged following BCAS surgery (Figs. S4a–c).

To investigate the potential link between loss of cholinergic innervation and increased RGMa expression in the hippocampus, we tested whether hippocampus-projecting cholinergic neurons express neogenin. Retrograde tracing was performed by injecting RetroBeads into the dentate gyrus (Fig. S4d). This procedure revealed that a subset of cells in the MS/DBB were positive for RetroBeads. Furthermore, some of these RetroBead-positive cells were also immunopositive for both ChAT and neogenin (Fig. S4e). These results suggest that cholinergic neurons projecting to the hippocampus in the MS and DBB express neogenin.

Finally, we assessed the potential therapeutic impact of the anti-RGMa antibody on the loss of cholinergic innervation. We found that the decreased ChAT-positive area within the dentate gyrus after BCAS surgery was ameliorated by the anti-RGMa antibody (Fig. 6d–f). Together, we speculated that the anti-RGMa neutralizing antibody attenuated BCAS-induced cognitive impairment by improving cholinergic innervation disturbance as well as neurogenesis impairment.

## Discussion

In the present study, we provide new insights into the molecular pathology in the hippocampus of a mouse model of VaD and propose anti-RGMa treatment as a novel therapeutic strategy to counteract cognitive impairment associated with VaD.

Hippocampal neurogenesis, characterized by the production of new neurons from neural stem cells within the SGZ of the dentate gyrus, is integral to learning, memory, and emotion regulation [12,13]. A decline in this process contributes to the cognitive deficits observed in Alzheimer's disease and aging [17,18]. In our study, at five weeks post-BCAS surgery, we observed a reduction in DCX-positive immature neurons, indicating impaired neurogenesis, primarily attributed to the excessive death of newborn neurons. Considering the stepwise progression of hippocampal neurogenesis, which includes the proliferation, subsequent differentiation, and maturation of neural stem cells into mature neurons [11,54], two key factors may contribute to the observed reduction in DCX-positive immature neurons: a decrease in the total number or proliferation rate of neural stem cells, or an impairment in the survival of newly born neurons. Notably, although BCAS surgery may influence other types of neural cells besides DCX-positive immature neurons in the hippocampus, we found no significant variations in NeuN-positive cells in the GCL or in Sox2-positive cells in the SGZ. Furthermore, the administration of BrdU to mice three weeks post-surgery resulted in an initial increase in Sox2/BrdU-positive cells, demonstrating an active proliferative response of neural stem cells. However, this initial increase was followed by a reduction in the number of DCX/BrdU double-positive cells two weeks after BrdU administration. In addition, after BCAS surgery, the number of apoptotic cells in the SGZ increased noticeably. These results suggest that while BCAS surgery does not inhibit the initial generation of newborn neurons, it detrimentally impacts their survival during the differentiation phase.

In our previous work, we explored the role of RGMa in neurogenesis under physiological conditions, demonstrating that RGMa suppression enhanced adult hippocampal neurogenesis [38]. Additionally, other studies have reported that RGMa expression significantly increased following ischemia-reperfusion in a middle cerebral artery occlusion/reperfusion (MCAO/R) model in mice, and upregulation of RGMa was also observed in primary culture neurons exposed to oxygen-glucose deprivation/reoxygenation (OGD/R) [55]. These insights prompted us to explore whether RGMa expression also increases under hypoxic conditions caused by CCH. As expected, we found that RGMa expression was upregulated in BCAS-treated mice from one week post-surgery.

**Fig. 7:**
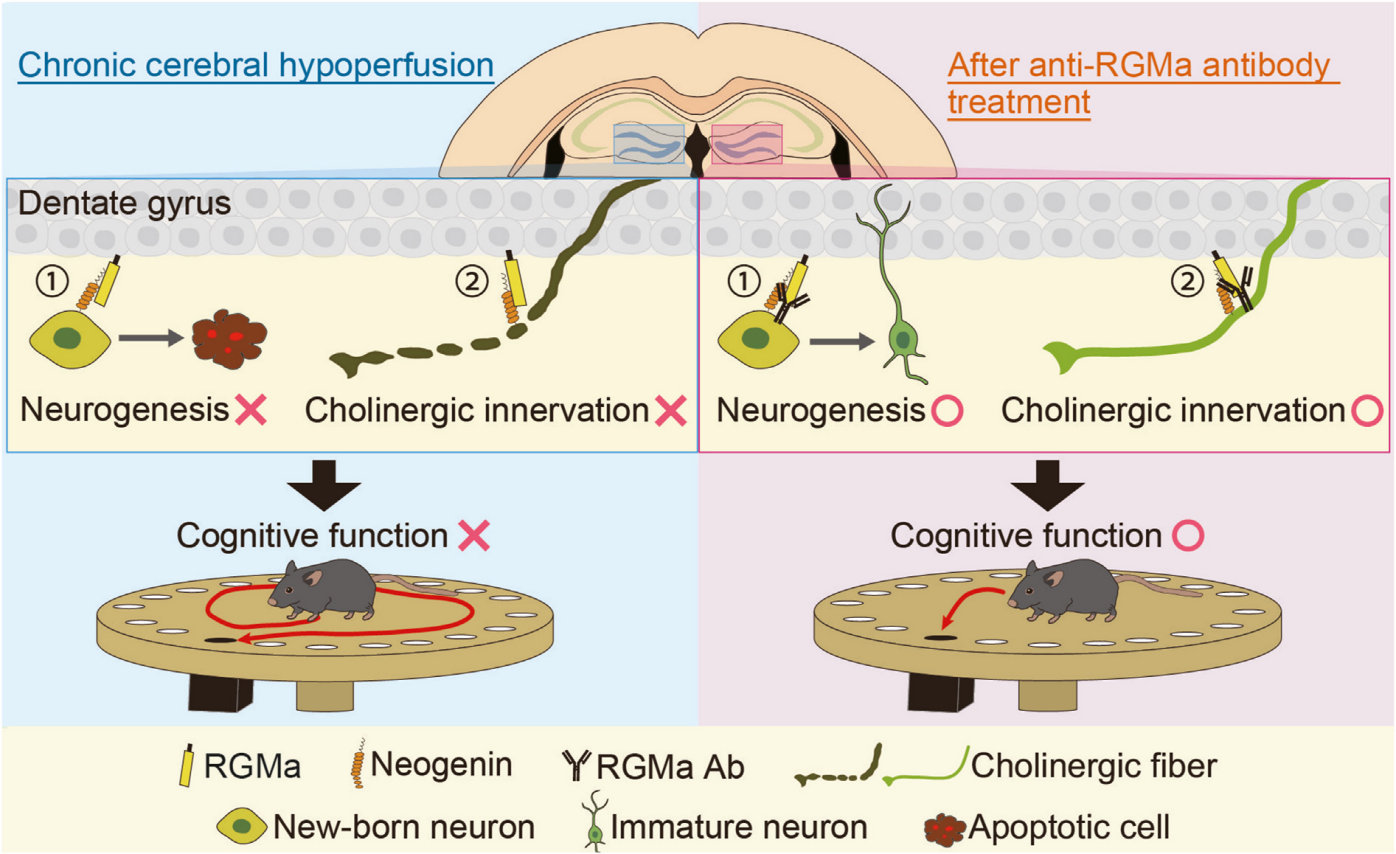
The proposed mechanisms of anti-RGMa antibody treatment on the pathophysiology of BCAS model mice. Left: RGMa levels are elevated in the dentate gyrus of BCAS model mice. RGMa binds to its receptor, neogenin, on young adult-born neurons, promoting their apoptosis and disrupting their maturation into mature neurons. RGMa also interacts with the cholinergic axon via its receptor Neogenin, compromising cholinergic innervation. Right: Treatment with the anti-RGMa antibody prevents the apoptosis of young adult-born neurons and denervation of cholinergic fibers by prevention of RGMa-neogenin signaling, leading to the improvement of cognitive dysfunction. BCAS, bilateral common carotid artery stenosis; RGMa, Repulsive Guidance Molecule A.

Anti-RGMa antibody treatment drastically ameliorated BCAS-induced impairment of hippocampal neurogenesis, alleviated the reduction in DCX-positive cells, and promoted the survival and maturation of newborn neurons. Considering the ability of the antibody to penetrate blood-brain barrier (BBB) under BCAS conditions, we confirmed the presence of the anti-RGMa antibody in the hippocampus by staining for human IgG (Fig. 4b), demonstrating that the antibody can indeed cross the BBB. Generally, the permeability of standard antibodies across the BBB is around 0.1–1% (brain/circulating antibodies) [56,57]. Immunohistochemical analyses revealed that RGMa was primarily expressed in mature neurons in the GCL, whereas neogenin was primarily expressed in DCX-positive differentiating neurons in the SGZ. This suggests a potential direct interaction between RGMa and neogenin in differentiating neurons. Previous studies from our group have demonstrated that the treatment of hippocampal neural stem cells expressing neogenin with recombinant RGMa protein in vitro increased apoptosis without any change in their proliferation rates [38]. Notably, RGMa-induced apoptosis was mitigated when neogenin was functionally blocked using shRNA. Therefore, one proposed mechanism by which the anti-RGMa antibody improves neurogenesis and alleviates subsequent cognitive impairment is its ability to prevent apoptosis in differentiating neurons by inhibiting the RGMa-neogenin pathway (Fig. 7).

RGMa and its receptor, neogenin, play pivotal roles in a variety of physiological processes and are expressed in numerous cell types [20]. Consequently, anti-RGMa antibodies may improve cognitive impairment through various contributing factors. Considering the hippocampal pathology, the reduction of acetylcholine and cholinergic markers stands out as a significant feature of VaD pathology [51,52], which is considered to be involved in the pathophysiology of cognitive impairment. Furthermore, previous in vitro and in vivo studies have demonstrated the inhibitory effect of RGMa on neurite outgrowth and have shown that anti-RGMa antibodies can counteract axonal degeneration by disrupting the RGMa-neogenin signaling [22,31,58]. Based on these insights, we hypothesized that anti-RGMa antibody treatment alleviates cholinergic fiber loss induced by chronic hypoperfusion. As expected, cholinergic innervation within the hippocampus was significantly decreased, and administration of the anti-RGMa antibody significantly ameliorated the impaired cholinergic innervations in the BCAS mice. Regarding the mechanism, we speculate that the anti-RGMa antibody may inhibit axonal degeneration by preventing RGMa-neogenin signaling in cholinergic fibers (Fig. 7). This is supported by two key observations: first, cholinergic neurons projecting from the MS and DBB to the hippocampus were found to express neogenin; second, the number of cholinergic neuron cell bodies in the MS and DBB was not affected by BCAS surgery, indicating that it is primarily the cholinergic fibers within the hippocampus that are compromised, rather than the cholinergic neurons themselves. These insights collectively strengthen our hypothesis and highlight the potential of the anti-RGMa antibody treatment for preserving cholinergic integrity in the hippocampus under chronic hypoperfusion conditions.

Patients with VaD are known to exhibit disturbances in cognitive functions that rely on the hippocampus and white matter [1]. While our BCAS model mice exhibited white matter lesions as well as hippocampal pathology, increased RGMa expression and therapeutic effects of the anti-RGMa antibody, as detected by IHC analysis, were observed only in the hippocampus. In line with this, the behavioral experiments showed that treatment with the anti-RGMa antibody ameliorated cognitive dysfunctions such as spatial learning/memory and novel object recognition, in which hippocampal neurogenesis is considered crucial [59]. Collectively, these findings indicate that RGMa expression is associated with CCH-induced cognitive decline by enhancing hippocampal pathology rather than white matter pathology, and that the anti-RGMa antibody exerts its therapeutic effect by normalizing hippocampal neurogenesis and cholinergic innervation.

The present study, while elucidating the therapeutic potential of the anti-RGMa antibody in a VaD model, had several limitations. First, anti-RGMa antibodies influence various cellular processes, including promoting neurite outgrowth, facilitating remyelination, attenuating immune responses, and stimulating angiogenesis [60]. Given this multifaceted impact, it is imperative to investigate the broader therapeutic effects on BCAS-induced pathologies. In particular, the influence of antibodies on BBB disturbances, a hallmark of the BCAS model [1], warrants detailed exploration in the context of neurogenic and cognitive recovery.

Second, the underlying mechanisms of RGMa upregulation and whether the expression of RGMa is altered in the hippocampus of patients with VaD have not yet been clarified. In future studies, these points need to be determined.

Finally, to address the pathomechanism of VaD, we employed the BCAS mouse model because it presents milder hypoperfusion than other available models [37,49]. An important limitation of the BCAS method is that it induces a sudden decline in cerebral perfusion immediately after the surgery [37], which differs from the slowly progressive chronic hypoperfusion observed in patients with carotid stenosis. This divergence poses challenges to understanding the effects of chronic hypoperfusion. Moreover, as VaD primarily affects the geriatric population [61], further studies are needed to explore whether the RGMa neutralizing antibody has therapeutic effects in aged mice that have undergone BCAS surgery.

In conclusion, our research marks a pioneering effort to develop a novel therapeutic agent for VaD using an anti-RGMa antibody. As disruptions in both neurogenesis and cholinergic fibers are prevalent in numerous CNS diseases, understanding the role of RGMa is becoming increasingly important. Future studies should delve deeper into the RGMa's potential implications and its applicability across a broader spectrum of pathological conditions, thereby emphasizing the potential of this research direction.

## Authors’ Contributions

T.I. and T.Y. conceived the study. M.Y., T.I., and T.Y. designed the experiments. M.Y. conducted the experiments and analyzed the data. H.U., T.M., and N.S. contibuted the methodology. M.Y., T. I., and T. Y. wrote the manuscript.

## Ethics statement

All experimental procedures were approved by the Institutional Animal Care Committee of Osaka University and complied with the guidelines for the care and use of laboratory animals of Osaka University.

## Date availability

All data generated and analyzed during this study are included in this published article (and its supplementary information files) and available from the corresponding author upon reasonable request.

## Funding statement

This work was supported by JSPS KAKENHI (grant number 21H05049 to T.Y.) and AMED-CREST (grant number 23gm1210005h0006 to T.Y.).

## Declaration of competing interest

A humanized anti-RGMa monoclonal antibody (unasnemab/MT-3921) was developed by Osaka University, Chiba University, and Mitsubishi Tanabe Pharma Corporation and are the property of them. The Department of Neuro-Medical Science was co-founded by Osaka University and Mitsubishi Tanabe Pharma Corporation.
