# Supplementary material for: Anti-RGMa neutralizing antibody ameliorates vascular cognitive impairment in mice
Source: Neurotherapeutics. 2024 Nov 29;22(2):e00500. doi: 10.1016/j.neurot.2024.e00500 (PMC12014345; doi:10.1016/j.neurot.2024.e00500)
Supplement: Multimedia component 1 [file mmc1.pdf]

## Fig. S1

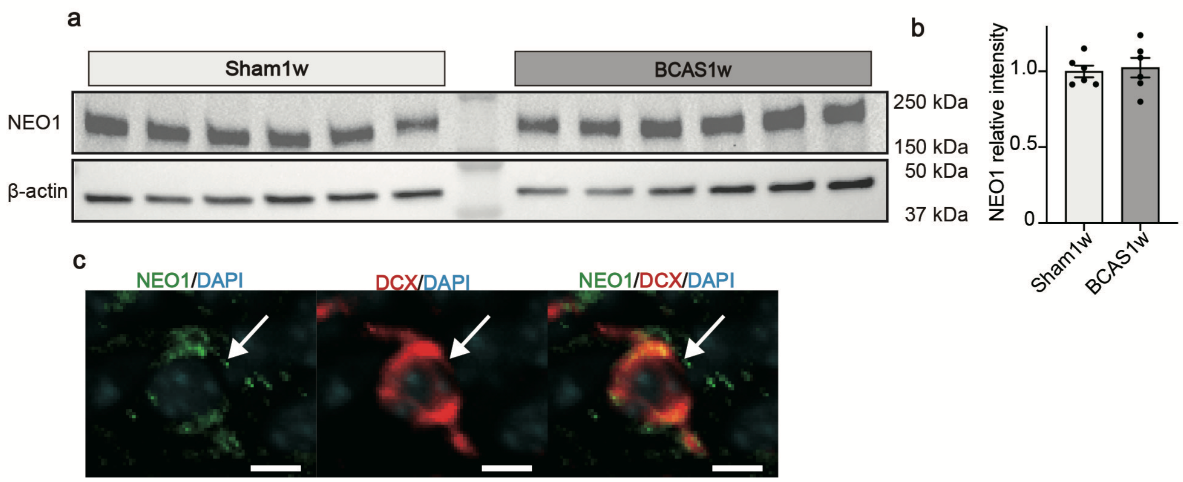


**Fig. S1 Neogenin expression in the hippocampus of BCAS model and control mice.**

**a** Western blot for NEO1 in the hippocampus 1 weeks post-surgery.

**b** Relative western blot density quantification of NEO1 in the hippocampus 1 weeks post-surgery. Mean ± SEM; n = 6 (sham1w), n = 6 (BCAS1w).

**c** Representative images of DCX and Neogenin (NEO1) staining in the subgranular zone of adult control mice. Nuclei were stained with DAPI. Scale bars: 5 µm.

## Fig. S2

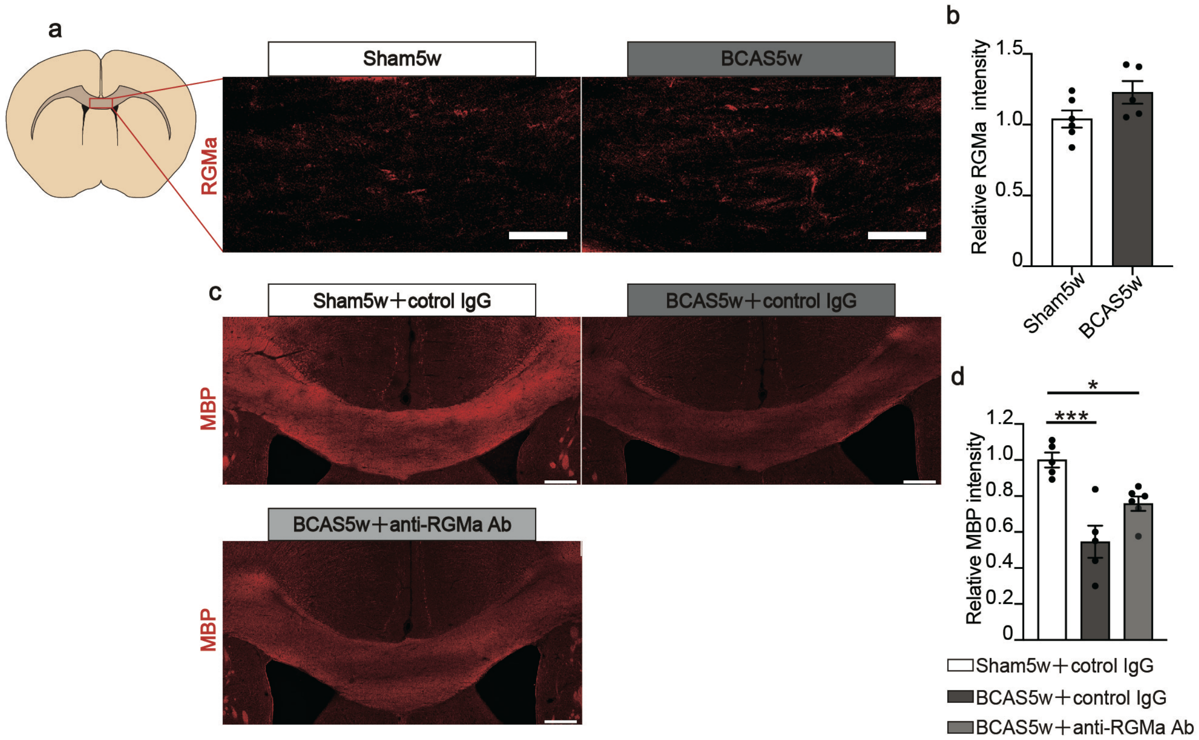


**Fig. S2 The RGMa expression and white matter lesions in the corpus collosum of the BCAS model mouse.**

**a** The left illustration highlights the specific region where representative images were captured, displaying RGMa staining in the corpus callosum at 5 weeks after the surgery. Scale bars: 100 µm.

**b** Quantitative data displaying the relative RGMa intensity in the corpus callosum at 5 weeks post-surgery. Mean ± SEM; n = 6 (sham5w), n = 5 (BCAS5w); unpaired Student's t-test.

**c** Representative images of MBP staining in the corpus callosum at 5 weeks post-surgery. Scale bars: 100 µm.

**d** The graph on the right is quantitative data displaying the relative MBP intensity in the corpus callosum at 5 weeks post-surgery. Mean ± SEM; n = 5 (sham5w+control IgG), n = 5 (BCAS5w+control IgG), n = 6 (BCAS5w+anti-RGMa Ab); one-way ANOVA with Tukey's multiple comparisons test: sham5w+control IgG vs BCAS5w+control IgG: ***$p < 0.001$, sham5w+control IgG vs BCAS5w+anti-RGMa Ab: *$p < 0.05$.

## Fig. S3

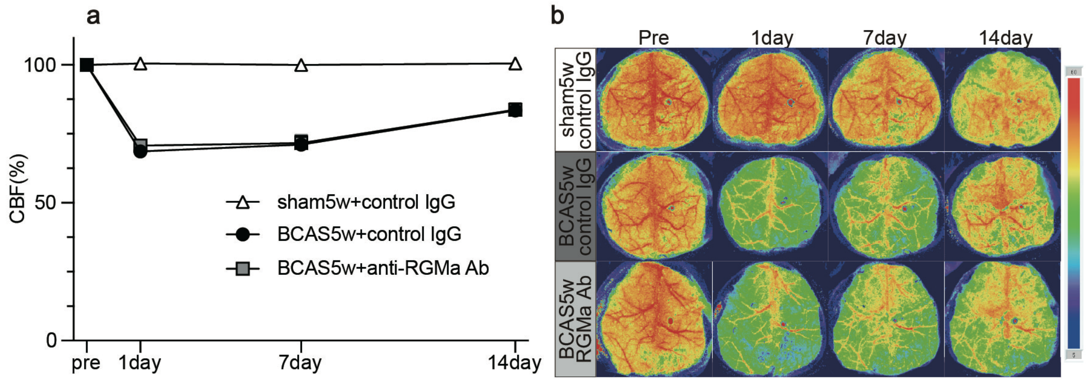


**Fig. S3 The effect of RGMa neutralizing antibody on CBF decline caused by BCAS surgery.**

**a** Temporal changes in CBF relative to baseline after BCAS or sham surgery. Sample sizes: pre, day 1 and day 7: n = 18 (sham5w+control IgG), n = 12 (BCAS5w+control IgG), n = 13 (BCAS5w+anti-RGMa Ab); day 14: n = 15 (sham5w+control IgG), n = 9 (BCAS5w+control IgG), n = 13 (BCAS5w+anti-RGMa Ab).

**b** Representative images from laser speckle flowmetry before BCAS or Sham surgery (pre) and on days 1, 7, and 14 post-surgery.

## Fig. S4

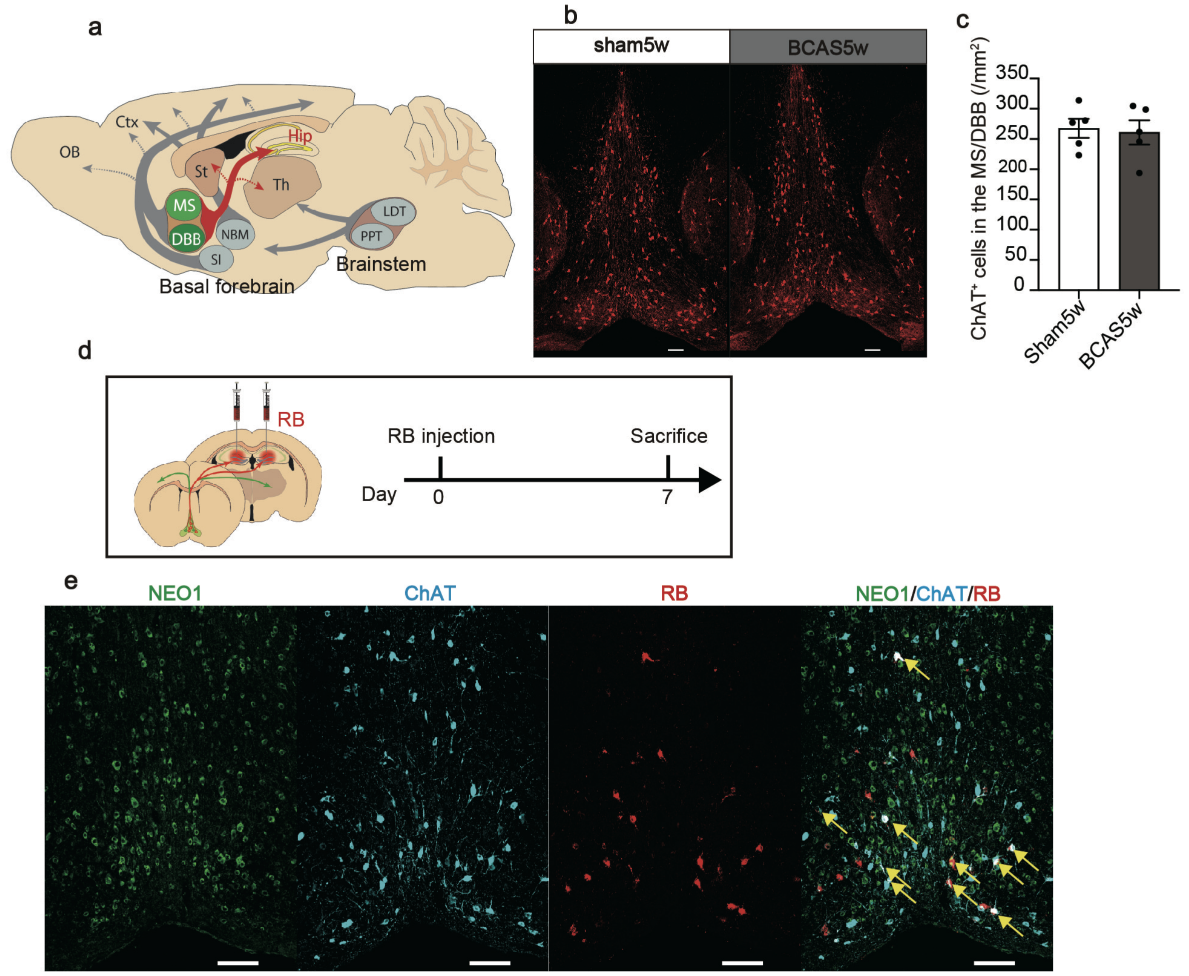


**Fig. S4 The cell bodies of cholinergic neurons in the MS/DBB of the BCAS model mice.**

**a** An illustrated overview providing insight into the cholinergic pathway. In the basal forebrain, cholinergic projections include the medial septum (MS), diagonal band of Broca (DBB), nucleus basalis of Meynert (NBM), and substantia innominata (SI). Notably, the MS and DBB are the specific sites where cholinergic neurons project to the hippocampus. In contrast, the brainstem's cholinergic projections are comprised of the laterodorsal tegmental (LDT) and pedunculopontine tegmental nuclei (PPT). Adapted from [Paul S, et al], [2015], [*Front Aging Neurosci*], [7].(1)

**b** Representative images depicting ChAT staining in the medial septum and the diagonal band of Broca, taken 5 weeks post-surgery. Scale bars: 100 µm.

**c** Quantitative data displaying the number of ChAT-positive cells in the medial septum and the diagonal band of Broca at 5 weeks post-surgery. Mean ± SEM; n = 5 (sham5w), n = 5 (BCAS5w); unpaired Student's t-test.

**d** Experimental timeline for retrograde tracing using Retrobeads: A fluorescent retrograde tracer (red RetroBeads from Lumafluor) was stereotactically injected into the dentate gyrus

of both hippocampi in a control adult mouse. Seven days post-injection, the MS and the DBB were examined for the transfer of the fluorescent label.

**e** Representative images displaying co-localization of Neogenin, ChAT, and Retrobeads in the MS and the DBB of a control adult mouse brain. Cells exhibiting positivity for all three markers are highlighted by yellow arrows. Scale bars represent 100 μm.

**References**

1. Mohapel P, Leanza G, Kokaia M, Lindvall O. Forebrain acetylcholine regulates adult hippocampal neurogenesis and learning. Neurobiol Aging. 2005;26(6):939–46.
